# Application of standardised effect sizes to hospital discharge outcomes for people with diabetes

**DOI:** 10.1186/s12911-020-01169-z

**Published:** 2020-07-07

**Authors:** Tim Robbins, Sarah N. Lim Choi Keung, Sailesh Sankar, Harpal Randeva, Theodoros N. Arvanitis

**Affiliations:** 1 Institute of Digital Healthcare, International Digital Laboratory, WMG, University of Warwick, Coventry, CV4 7AL UK; 2 Warwickshire Institute for the Study of Diabetes, Endocrinology & Metabolism, University Hospitals Coventry & Warwickshire NHS Trust, Clifford Bridge Road, Coventry, CV2 2DX UK

**Keywords:** Effect size, Readmission, Mortality, Diabetes

## Abstract

**Background:**

Patients with diabetes are at an increased risk of readmission and mortality when discharged from hospital. Existing research identifies statistically significant risk factors that are thought to underpin these outcomes. Increasingly, these risk factors are being used to create risk prediction models, and target risk modifying interventions. These risk factors are typically reported in the literature accompanied by unstandardized effect sizes, which makes comparisons difficult. We demonstrate an assessment of variation between standardised effect sizes for such risk factors across care outcomes and patient cohorts. Such an approach will support development of more rigorous risk stratification tools and better targeting of intervention measures.

**Methods:**

Data was extracted from the electronic health record of a major tertiary referral centre, over a 3-year period, for all patients discharged from hospital with a concurrent diagnosis of diabetes mellitus. Risk factors selected for extraction were pre-specified according to a systematic review of the research literature. Standardised effect sizes were calculated for all statistically significant risk factors, and compared across patient cohorts and both readmission & mortality outcome measures.

**Results:**

Data was extracted for 46,357 distinct admissions patients, creating a large dataset of approximately 10,281,400 data points. The calculation of standardized effect size measures allowed direct comparison. Effect sizes were noted to be larger for mortality compared to readmission, as well as for being larger for surgical and type 1 diabetes cohorts of patients.

**Conclusions:**

The calculation of standardised effect sizes is an important step in evaluating risk factors for healthcare events. This will improve our understanding of risk and support the development of more effective risk stratification tools to support patients to make better informed decisions at discharge from hospital.

## Background

Increasing numbers of hospital inpatients have a co-existent diagnosis of diabetes [1]. These patients are at an increased risk of both readmission [2] and mortality [3, 4]. Multiple studies have been performed aiming to identify statistically significant risk factors for poor outcomes, when patients with diabetes are discharged from hospital [5, 6]. These studies typically identify risk factors for generalised populations of inpatients with diabetes [7, 8], or for an individual specific cohorts who may have been admitted for a particular condition or group of conditions [9–11]. Almost universally, these studies report statistically significant risk factors for an individual outcomes (either readmission or mortality) and report unstandardized effect size measures, usually as odds ratios.

The use of unstandardized effect size measures that report only on individual outcomes for individual patient cohorts, makes effect size comparisons between groups difficult. Comparisons between studies are additionally difficult as, unlike clinical trials, extracted electronic health record data is rarely made available as supplementary material to research articles, due to the risk of inadvertently compromising anonymity of the data. Furthermore, whilst standardised effect sizes can be calculated if both the sample size and standard deviation are given with unstandardised effect statistics in articles, it is recognised that this information is too often incomplete and can be a laborious process across multiple studies even if it is available [12].

This represents a key challenge in identifying optimal targets and outcome measures for the delivery of interventions to improve the discharge process from hospital for patients with diabetes. In particular, attempts to create risk stratification tools for patients with diabetes at discharge have only had limited success, often reporting only moderate area under the curve (AUC) values and restricted predictive values [13, 14].

Standardised effect size calculations allow the direct comparison between risk factors, outcomes and cohorts. Similar to other statistical tests, there are a range of effect size statistics available, with well over 60 reported in the literature [15]. The appropriate standardised effect size statistic needs to be selected relevant to the variables in question, with *d* statistics (such as Cohen’s *d or*) appropriate for continuous dependant variables & predictors, whilst differing tests are relevant for effect size estimates related to categorical date (such as Phi or Cramér’s V) [16].

Effect sizes are descriptive statistics that support both clinicians and researchers to interpret study findings. The interpretation of effect sizes can be done in isolation against pre-defined published levels of effect or “rules of thumb” [17]. Table 1 demonstrate published rules of thumb for Cohen’s d [18] and Phi statistics [19, 20]. Importantly however, the importance of any effect is dependent on what is being studied, with for instance very small effect sizes being important in certain circumstances (for example life threating situations) [21]. Effect sizes statistics are also particularly valuable when looking to make comparisons, for example between different predictors, cohorts or variables and in is primarily in this context that standardised effect sizes have utility in considering risk predictors for negative clinical outcomes.

**Table 1:** “Rules of thumb for interpreting effect sizes”

Cohen’s ***d***		Phi Coefficent	
Effect Size	***d*** value	Effect Size	***d*** value
Very small	0.01	Negligible	0.00 to < 0.10
Small	0.2	Weak	0.10 to < 0.20
Medium	0.5	Moderate	0.20 to < 0.40
Large	0.8	Relatively strong	0.40 to < 0.60
Very large	1.2	Strong	0.60 to < 0.80
Huge	2	Very strong	0.80 to < 1.00

In this study, we extract data from a large tertiary referral centre in order to calculate standardised effect sizes for pre-specified risk factors, across outcome measures and across patient cohorts. This research demonstrates the importance of calculating standardised effect sizes, a practice more typical in the psychological literature than medical literature. It further demonstrates important variation in risk at discharge from hospital for patients with diabetes.

## Methods

The study adopted a retrospective evaluation of data extracted from electronic health record (EHR) of a large tertiary referral centre, in the West Midlands region of the United Kingdom, for all adult patients discharged from University Hospitals Coventry and Warwickshire NHS Trust with a diagnosis of diabetes, over a 3-year period. Data were extracted for an exemplar set of 10 pre-specified risk factor variables, these variables were selected based on both pre-specification from the published research literature [22], and the ease of which data for these variables can be extracted from inpatient electronic health records. Ease of extraction considered to ensure the results are generalizable to other healthcare organisations internationally. The selected extracted variables and are listed in Table 2. Outcome variable data were extracted for hospital readmission within 30 days and mortality within 180 days of hospital discharge.

**Table 2:** Readily extractable pre-specified risk factors

**Selected Risk Factors**	
Age	
Sex	
Co-morbidity burden	
Previous DKA	
Dementia	
DSN review	
T1DM	
T2DM patients	
Unknown diabetes type	
Weekend Discharged	

The diagnosis of diabetes was taken from the coding of patients at discharge and, thus, if there was discrepancy in the diagnosis within the record, the latest diagnosis of diabetes at discharge was used. Maternity patients were excluded from the study, due to the differing nature of maternity care and readmission patterns. Patients discharged within the last 6 months of the study period were not evaluated as index patients, to ensure that all patients had a full period of 6 months follow up on the electronic health record to assess for the outcome measures of interest.

The association between risk factor variables and outcomes of interest was analysed using Chi Squared Tests for categorical variables and Student’s t-Test for continuous variables, following adequate assessment for skew and kurtosis to ensure normality. an absolute skew value larger than 2 or an absolute kurtosis (proper) larger than 7 may be used as reference values for determining substantial non-normality [23].

A *p*-value of < 0.05 was considered significant. Standardised size was evaluated using Phi coefficient for categorical variables and Cohen’s D for continuous variables. The statistical significance and effect size was evaluated for the following patient cohorts: all patients discharged with a diagnosis of diabetes; all emergency admissions discharged with a diagnosis of diabetes; all emergency admissions discharged with a diagnosis of Type 2 Diabetes; all emergency admissions discharged with a diagnosis of type 1 diabetes; all elective admissions discharged with a diagnosis of diabetes; all elective admissions discharged with a diagnosis of type 2 diabetes; all elective admissions discharged with a diagnosis of type 2 diabetes; all patients with diabetes discharged from surgical care; all patients with type 1 diabetes discharged from surgical care; and all patients discharged with type 2 diabetes from surgical care.

All statistical testing was performed using Microsoft Excel 2016 and IBM’s SPSS v24.

Ethical approval was granted by the local NHS Trust Research Ethics Committee, at University Hospitals Coventry & Warwickshire NHS Trust through the Governance arrangements for Research Ethics Committee Process [Study Ref: GF0220 & GF0335]. Approval was also granted through the University of Warwick’s Biomedical & Scientific Research Ethics Committee [Study Ref: REGO-2017-2114].

## Results

Data was extracted for 46,367 distinct patient episodes resulting in discharge from hospital, over the study period, with a diagnosis of diabetes. Table 3 demonstrates the number of patients in each cohort. Table 4 illustrates the statistical significance of each risk factor in relation to readmission per patient cohort, separated into categorical risk factor variables (evaluated with the Chi Squared Test) and continuous variables (evaluated using student’s t-test) for readmission. Table 5 similarly demonstrates statistical significance testing for mortality. Table 6 illustrates the standardised effect sizes of each risk factor related to readmission per patient cohort, separated again into categorical risk factors (evaluated using Phi coefficient) and continuous risk factors (evaluated using Cohen’s D). Table 7 illustrates effect sizes in relation to mortality at 180 days.

**Table 3:** Patient cohorts and sample size

Patient Cohort	Number of patients in sample
All diabetes discharges	46,367
Emergency admission discharges with diabetes	20,140
Elective admission discharges with diabetes	23,379
Emergency admission surgical discharges	3032
Emergency admission medical discharges	14,250
All surgical care discharges with type 1 diabetes	399
All surgical care discharges with type 2 diabetes	2547
All medical care discharges with type 1 diabetes	1455
All medical care discharges with type 2 diabetes	12,498

**Table 4:** Assessment of statistically significant association between risk factors and readmission at 30 days. [Shaded cells reflect lack of statistical significant value of *p* < 0.05]

Assessment of statistically significant association between risk factors and readmission at 30 days.	Statistical Test	All diabetes discharges	Emergency admission discharges	Elective admission discharges	Emergency admission surgical discharges	Emergency admission medical discharges	T1DM Surgical patients	T2DM Surgical patients	T1DM Medical patients	T2DM Medical patients
Age	TTEST	< 0.01	0.33	< 0.01	0.09	0.08	< 0.01	0.6	0.07	0.94
Sex	CHISQ	0.01	0.04	0.62	0.06	0.39	< 0.01	0.42	< 0.01	0.16
Co-morbidity burden	TTEST	< 0.01	< 0.01	< 0.01	0.56	< 0.01	< 0.01	< 0.01	0.07	< 0.01
Previous DKA	CHISQ	< 0.01	< 0.01	< 0.01	< 0.01	0.68	< 0.01	0.57	0.19	0.75
Dementia	CHISQ	< 0.01	< 0.01	0.05	0.09	< 0.01	0.27	0.12	0.34	0.02
DSN review	CHISQ	< 0.01	< 0.01	< 0.01	0.18	< 0.01	0.05	0.38	< 0.01	< 0.01
T1DM	CHISQ	< 0.01	< 0.01	< 0.01	< 0.01	0.03	NA	NA	NA	NA
T2DM patients	CHISQ	< 0.01	0.10	< 0.01	0.06	0.46				
Unknown diabetes type	CHISQ	< 0.01	< 0.01	< 0.01	0.15	< 0.01				
Weekend Discharged	CHISQ	< 0.01	0.06	< 0.01	0.01	< 0.01	0.44	0.03	0.29	0.36

**Table 5:** Assessment of statistically significant association between risk factors and mortality at 180 days. . [Shaded cells reflect lack of statistical significant value of *p* < 0.05]

Assessment of statistically significant association between risk factors and mortality at 180 days.	Statistical Test	All discharges	Emergency admission discharges	Elective admission discharges	Emergency admission surgical discharges	Emergency admission medical discharges	T1DM Surgical patients	T2DM Surgical patients	T1DM Medical patients	T2DM Medical patients
Age	TTEST	< 0.01	< 0.01	< 0.01	< 0.01	< 0.01	< 0.01	< 0.01	< 0.01	< 0.01
Sex	CHISQ	< 0.01	< 0.01	< 0.01	< 0.01	< 0.01	0.92	0.60	0.07	< 0.01
Co-morbidity burden	TTEST	< 0.01	< 0.01	< 0.01	< 0.01	< 0.01	< 0.01	< 0.01	< 0.01	< 0.01
Previous DKA	CHISQ	< 0.01	< 0.01	< 0.01	0.20	< 0.01	0.01	0.65	< 0.01	< 0.01
Dementia	CHISQ	< 0.01	< 0.01	0.40	< 0.01	< 0.01	< 0.01	0.01	< 0.01	< 0.01
DSN review	CHISQ	< 0.01	0.02	< 0.01	< 0.01	< 0.01	0.80	0.05	< 0.01	0.31
T1DM	CHISQ	< 0.01	0.09	0.14	0.34	< 0.01	NA	NA	NA	NA
T2DM patients	CHISQ	< 0.01	< 0.01	0.01	< 0.01	< 0.01				
Unknown diabetes type	CHISQ	0.7	0.43	< 0.01	0.09	0.82				
Weekend Discharged	CHISQ	< 0.01	< 0.01	< 0.01	< 0.01	< 0.01	0.58	0.34	0.12	< 0.01

**Table 6:** Assessment of standardised effect sizes for statistically significant risk factors for readmission at 30 days. [Shaded cells did not reach statistical significant in Table 4]

Assessment of standardised effect size between risk factors and readmission at 30 days.	Statistical Test	All diabetes discharges	Emergency admission discharges	Elective admission discharges	Emergency admission surgical discharges	Emergency admission medical discharges	T1DM Surgical patients	T2DM Surgical patients	T1DM Medical patients	T2DM Medical patients
Age	Cohens D	0.08		0.16			0.45			
Sex	Phi	−0.01	− 0.01				− 0.10		0.07	
Co-morbidity burden	Cohens D	− 0.18	− 0.11	− 0.37		− 0.12	0.39	− 0.17		− 0.18
Previous DKA	Phi	− 0.10	− 0.03	− 0.10	− 0.08		− 0.17			
Dementia	Phi	0.03	0.03	0.01		0.03				0.06
DSN review	Phi	−0.03	0.04	0.02		0.07			−0.11	0.12
T1DM	Phi	−0.06	−0.03	− 0.05	−0.06	− 0.02	NA	NA	NA	NA
T2DM patients	Phi	0.02		0.03						
Unknown diabetes type	Phi	0.03	0.03	0.03		0.03				
Weekend Discharged	Phi	−0.01		0.03	0.05	− 0.03		0.04

**Table 7:** Assessment of standardised effect size for statistically significant risk factors for mortality at 180 days [Shaded cells did not reach statistical significance in Table

Assessment of standardised effect size between risk factors and mortality at 180 days	Statistical Test	All diabetes discharges	Emergency admission discharges	Elective admission discharges	Emergency admission surgical discharges	Emergency admission medical discharges	T1DM Surgical patients	T2DM Surgical patients	T1DM Medical patients	T2DM Medical patients
Age	Cohens D	−0.75	−0.71	−0.45	− 0.87	−0.69	−1.38	− 0.78	−1.64	−0.56
Sex	Phi	−0.03	−0.03	− 0.03	−0.03	− 0.03				−0.03
Co-morbidity burden	Cohens D	−0.70	−0.61	− 0.53	−0.85	− 0.57	−0.85	− 0.82	−1.27	−0.47
Previous DKA	Phi	0.05	0.08	0.03		0.09	0.13	0.01	0.23	0.03
Dementia	Phi	−0.06	−0.05		−0.04	− 0.05	−0.16	− 0.05	−0.09	− 0.04
DSN review	Phi	−0.04	0.02	−0.02	− 0.04	0.05			0.16	
T1DM	Phi	0.04	0.09			0.09	NA	NA	NA	NA
T2DM patients	Phi	−0.07	−0.08	−0.02	− 0.10	−0.08				
Unknown diabetes type	Phi			0.02						
Weekend Discharged	Phi	−0.02	< 0.01	0.02	−0.03	− 0.04				− 0.04

Statistically significant associations with readmission at 30 days were found for 46 cohort/risk factors combinations, with 61 statistically significantly associations for mortality at 180 days. Following expectations, the effect size of most risk factors individually on outcomes was small. However, there was significant variation in effect size between risk factors, cohorts and outcome measures. The mean average effect size for categorical values considering readmission (Phi Coefficient) was 0.05, with the mean average effect size for continuous variables being (Cohen’s D) 0.23. Mean average effect size for categorical values, considering mortality, was 0.06 (Phi coefficient) and for continuous variables 0.81 (Cohen’s D). Effect sizes were notably larger for surgical cohorts of patients, in particular surgical patients with T1DM.

## Discussion

We demonstrate that there is substantial variation in the effect sizes, regarding risk factors related to poor outcomes at discharge from hospital for patients with diabetes. Whilst a large number of candidate risk factors have been identified as statistically significant, there is variation in the effect sizes between individual risk factors. Typically, effect sizes for mortality were greater than effect sizes for readmission, suggesting that using the risk factors described here, it may be easier to predict risk related to mortality than readmission. This is particularly interesting, given that readmission is most commonly used as the maker of the success of the discharge process and a typical target for risk predication modelling and risk reduction interventions.

There is also substantial variation in both the statistical significance and effect size of individual risk factors between individual cohorts of patients with diabetes, as well as the overall combined effect sizes between individual patient cohorts. This suggests, again, that risk prediction may be easier for some cohorts of patients, particularly those with Type 1 Diabetes & those attending for surgery. The ability, with which we are able to predict risk from known risk factors, is important in the development and appropriateness of developing risk prediction tools, but also in targeting interventions to those most at need. The targeting of interventions, supported by evidence-based discussions of risk with patients, is essential to individualised sustainable healthcare.

This study does not aim to provide a comprehensive assessment of effect sizes for every patient cohort with diabetes at discharge from hospital, or for every known risk factor for poor outcomes. Rather, we demonstrate the substantial variations of standardised effect sizes between risk factors, outcomes and patient cohorts. It is important to note that there are a number of different effect size measures that could be used, Cohen’s D and Phi were selected as being widely used effect size measures and their suitability for use alongside statistical significance measures. This, therefore, lays important foundations for future research looking to explore individual risk factors, outcomes or cohorts in more depth, before possible future development of rigorous risk prediction models.

The study has a number of limitations. Firstly it is based only at a single centre, albeit with a large patient population over a significant period of time. Secondly, we have not attempted to control individual risk factors at this stage; this approach is however representative of the many studies identifying new risk factors and subsequently reporting with unstandardized effect sizes. It must be remembered that this is an exemplar paper demonstrating the utility of standardised effect sizes and the ability to predict risk may vary depending on other risk factors selected or cohorts analysed.

When considering the utility of standardised effect sizes it is notable that we have used two different effect size statistics (Cohen’s d & Phi coefficient), whilst the primary aim of using standardised effect size statistics is to enable comparison the outputs of the two statistical methods cannot be directly compared due to variations in the “rules of thumb” for their interpretation. Whilst some processes to enable conversion between effect size statistics have been published, there is no accepted approach to converting between all effect size statistics [16].

## Conclusions

We demonstrate the calculation of standardised effect sizes for risk factors related to poor outcomes when patients are discharge from hospital. Whilst individual effect sizes are often small, there is substantial variability between different risk factors, patient cohorts and outcomes. The use of standardised effect sizes allows the easier comparison between such groups, this in turn may facilitate the development of better risk stratification models and risk minimisation interventions. We hope that, as a consequence of this paper, more studies will look to calculate standardised effect sizes when considering risk factors, generating more directly comparable results and enabling more rapid translation into changes to patient care.

## Data Availability

Data was generated from the inpatient electronic health record patient-data of University Hospitals Coventry & Warwickshire NHS Foundation Trust. As is typical for data sets of this nature, whilst the information is anonymised, the ethical approval process requires analysis and storage of the raw data on secure NHS equipment due to the risk of inadvertent or indirect breeches to anonymization. The raw data may potentially be available from University Hospitals Coventry & Warwickshire NHS Trust subject to approval, ethical review and secure storage arrangements.

## Abbreviations

EHR: Electronic Health Record
NHS: National Health Service
T1DM: Type 1 Diabetes
T2DM: Type 2 Diabetes
